# Increased Cyclooxygenase-2-Derived Prostanoids Contributes to the Hyperreactivity to Noradrenaline in Mesenteric Resistance Arteries from Offspring of Diabetic Rats

**DOI:** 10.1371/journal.pone.0050593

**Published:** 2012-11-28

**Authors:** Fernanda E. Ramos-Alves, Diego B. de Queiroz, Juliana Santos-Rocha, Gloria P. Duarte, Fabiano E. Xavier

**Affiliations:** Departamento de Fisiologia e Farmacologia, Centro de Ciências Biológicas, Universidade Federal de Pernambuco, Recife, Brazil; The Chinese University of Hong Kong, Hong Kong

## Abstract

This study analyzed the effect of *in utero* exposure to maternal diabetes on contraction to noradrenaline in mesenteric resistance arteries (MRA) from adult offspring, focusing on the role of cyclooxygenase (COX)-derived prostanoids. Diabetes in the maternal rat was induced by a single injection of streptozotocin (50 mg/kg body weight) on day 7 of pregnancy. Contraction to noradrenaline was analyzed in isolated MRA from offspring of diabetic (O-DR) and non-diabetic (O-CR) rats at 3, 6 and 12 months of age. Release of thromboxane A_2_ (TxA_2_) and prostaglandins E_2_ (PGE_2_) and F_2α_ (PGF_2α_), was measured by specific enzyme immunoassay kits. O-DR developed hypertension from 6 months of age compared with O-CR. Arteries from O-DR were hyperactive to noradrenaline only at 6 and 12 months of age. Endothelial removal abolished this hyperreactivity to noradrenaline between O-CR and O-DR. Preincubation with either the COX-1/2 (indomethacin) or COX-2 inhibitor (NS-398) decreased noradrenaline contraction only in 6- and 12-month-old O-DR, while it remained unmodified by COX-1 inhibitor SC-560. In vessels from 6-month-old O-DR, a similar reduction in the contraction to noradrenaline produced by NS-398 was observed when TP and EP receptors were blocked (SQ29548+AH6809). In 12-month-old O-DR, this effect was only achieved when TP, EP and FP were blocked (SQ29548+AH6809+AL8810). Noradrenaline-stimulated TxB_2_ and PGE_2_ release was higher in 6- and 12-month-old O-DR, whereas PGF_2α_ was increased only in 12-month-old O-DR. Our results demonstrated that *in utero* exposure to maternal hyperglycaemia in rats increases the participation of COX-2-derived prostanoids on contraction to noradrenaline, which might help to explain the greater response to this agonist in MRA from 6- and 12-month-old offspring. As increased contractile response in resistance vessels may contribute to hypertension, our results suggest a role for these COX-2-derived prostanoids in elevating vascular resistance and blood pressure in offspring of diabetic rats.

## Introduction

Experimental and epidemiological studies have provided evidence that intrauterine exposure to maternal hyperglycemia produces several effects on offspring including insulin resistance, change in glucose metabolism and increased risk of hypertension in adult life [1]–[5]. This phenomenon termed “fetal programming” refers to the observation that an adverse environmental stimulus experienced *in utero* during the critical period of development correlates with a number of adult chronic diseases, including coronary heart disease, stroke and hypertension [6], [7]. In the offspring of diabetic rats, the hyperglycemia-programmed hypertension is age-dependent [1], [8], [9] and has been partially attributed to baroreflex dysfunction [10] and nephron deficiency [4], [8], [11]. In addition, some studies have suggested that functional change in the vasculature could also contribute to increase blood pressure on offspring of diabetic rats [3], [8]. These functional vascular changes are characterized by impaired endothelial function [8], [9], [12] and increased contractile responses to vasoconstrictor agents, such as noradrenaline and serotonin [2], [12]. Recently, we have demonstrated that exposure to maternal hyperglycemia is associated with age-dependent vascular cyclooxygenase (COX)-2 up-regulation and enhanced formation of contracting prostanoids, which impairs the endothelium-dependent relaxation in resistance arteries from adult offspring [9].

COX catalyzes conversion of arachidonic acid to prostanoids, which is known as important inflammatory response mediators. In addition, endothelial prostanoids production contributes to vascular tone regulation [13]. Two isoforms of COX have been identified in mammalian cells. COX-1 is constitutively expressed in most tissues, such as vascular endothelial cells, and is involved in homeostasis maintenance. Although COX-2 is expressed at low or undetectable level, it is readily upregulated by inflammatory, mitogenic and physical stimuli. Vascular COX-2 expression has been reported in several pathological conditions associated with cardiovascular risk, such as hypertension, diabetes, atherosclerosis, obesity and aging [13]–[20]. In addition to its contribution to impairment of the endothelium-dependent relaxations [9], [17], [19], COX-2-derived prostanoids have also been recognized as an important factor causing increased contraction in isolated arteries from hypertensive [15] and diabetic [18] rats. However, the impact of these endothelial factors on vascular contraction is specific to each experimental model or vascular bed studied. In this sense, we [19], [21] and others [22] have demonstrated that in resistance arteries from spontaneously hypertensive rats the COX-mediated impairment of endothelium-dependent relaxation is accompanied by uncharged noradrenaline-induced contraction. In resistance arteries from offspring of diabetic rats the contribution of these prostanoids to noradrenaline-induced contraction is still unknown. Once sympathetic vasoconstriction in resistance arteries is an important peripheral blood pressure regulator, this study has important implications with regard to arterial diameter regulation and blood pressure in offspring of diabetic rats.

In view of that, the present experiments were designed to study whether *in utero* exposure to maternal hyperglycemia alters contractile responses to noradrenaline in resistance arteries from adult offspring as well as the possible role of COX-2-derived prostanoids in this effect. Considering that in offspring of diabetic rats the endothelial function change with age [9], in the current study we also investigated the influence of age on noradrenaline responses.

## Methods

### Animals

All experimental procedures were approved by the Animal Care and Use Committee of the *Universidade Federal de Pernambuco* (approval reference number: 23076.015755/2008-96) and were conducted in accordance with the Guide for the Care and Use of Laboratory Animals published by the US National Institute of Health (NIH Publication No. 85-23, revised 1996).

Adult offspring of control (O-CR) and diabetic (O-DR) pregnant Wistar rats were studied. Diabetes mellitus in the maternal rat was induced experimentally by a single i.p. injection of streptozotocin (50 mg/kg body weight) on day 7 of pregnancy. The diabetes was confirmed by measuring plasma glucose concentrations (ACCU-CHEK®, Roche Diagnostics, Mannheim, Germany) on day 14 of pregnancy. Only pregnant females whose plasma glucose ranged between 250 and 500 mg/dL were included in the study. After birth, each litter was randomly reduced to six pups and restricted to male offspring only. The excess pups were sacrificed by CO_2_ inhalation followed by cervical dislocation. When the male number was not enough to complete six, females were used but discarded at weaning. All rats were housed at a constant room temperature (21°C), humidity (55%) and light cycle (12 h light/dark) and had free access to tap water and standard rat chow *ad libitum*. In this study we used O-CR and O-DR with 3, 6 and 12 months of age.

### Arterial Blood Pressure Measurement

Rats were anesthetized with ketamine, xylazine and acetopromazin mixture (64.9; 3.2 and 0.78 mg.Kg^−1^, respectively, *i.p.*). The right carotid artery was cannulated with a polyethylene catheter (PE-50) that was exteriorized in the mid scapular region. Adequacy of anaesthesia was assessed by monitoring withdrawal reflexes. After 24 hours, arterial pressure was measured in conscious, freely moving rats. The arterial cannula was connected to a transducer and pressure signals were recorded for a 60-min period using a data acquisition system model PowerLab 4/35 (ADInstruments Pty Ltd, Castle Hill, Australia).

### Vessel Preparation

Rats were anesthetized with ketamine, xylazine and acetopromazin mixture (64.9; 3.2 and 0.78 mg/Kg^−1^, respectively, *i.p.*) and killed by exsanguination. The mesenteric vascular bed was removed and placed in cold (4°C) Krebs Henseleit solution (KHS; in mmol/L: 115 NaCl, 2.5 CaCl_2_, 4.6 KCl, 1.2 KH_2_PO_4_, 1.2, MgSO_4_.7H_2_O, 25 NaHCO_3_, 11.1 glucose, and 0.03 EDTA). For reactivity experiments the third order branch of the mesenteric arcade was dissected and cut in segments of approximately 2 mm in length. Segments of mesenteric resistance arteries were mounted in a small vessel chamber myograph (Danish Myo Technology A/S, Aarhus, Denmark) to measure isometric tension according to the method described by Mulvany and Halpern [23]. After a 15-min equilibration period in oxygenated KHS at 37°C and pH 7.4, segments were stretched to their optimal lumen diameter for active tension development. Optimal lumen diameter was determined based on the internal circumference/wall tension ratio of the segments by setting the internal circumference, L_0_, to 90% of what the vessels would have if they were exposed to a passive tension equivalent to that produced by a transmural pressure of 100 mmHg [23]. Optimal lumen diameter was determined using specific software for normalization of resistance arteries (DMT Normalization Module; ADInstruments Pty Ltd, Castle Hill, Australia). Segments were washed with KHS and left to equilibrate for 30 min. Vessel contractility was then tested by an initial exposure to a high-K^+^ (120 mmol/L) solution.

### Experimental Protocols

After washout, segments were contracted with a concentration of noradrenaline that induced approximately 50% of the KCl contraction, and then acetylcholine (1 µmol/L) was added to assess the integrity of the endothelium. After 60 min, cumulative concentration–response curves for noradrenaline (10 nmol/L - 0.1 mmol/L) were generated. The role of the endothelium in the noradrenaline-induced contraction was evaluated in segments subjected to mechanical endothelium removal. The absence of endothelium was confirmed by the inability of acetylcholine (1 µmol/L) to induce relaxation and smooth muscle integrity was confirmed by the maintenance of the KCl- (120 mmol/L) induced contraction. The effect of the nonselective nitric oxide synthase inhibitor N-nitro-l-arginine methyl ester (L-NAME, 0.1 mmol/l) on concentration–response curves for noradrenaline was investigated.

The possible role of cyclooxygenase metabolites in noradrenaline-induced contraction was investigated in segments pre-incubated with either indomethacin (a COX-1 and COX-2 inhibitor, 10 µmol/L), [5-(4-chlorophenyl)-1-(4-methoxyphenyl)-3-trifluoromethylpyrazole] SC-560 (a COX-1 inhibitor, 1 µmol/L), N-(2-cyclohexyloxy-4-nitrophenyl) methansulfonamide (NS-398, a COX-2 inhibitor, 10 µmol/L), [1S-[1a,2a(Z),3a,4a]]-7-[3-[[2-(phenylamino) carbonyl]hydrazino]methyl]-7-oxabicyclo [2.2.1] hept-2-yl]-5-heptanoic acid (SQ29548, a TxA_2_ receptor (TP) antagonist, 1 µmol/L), 6-Isopropoxy-9-oxoxanthene-2-carboxylic acid (AH6809, a PGE_2_ receptor (EP_1_, EP_2_ and EP_3_) antagonist, 30 µmol/L) or 9α,15R-dihydroxy-11β-fluoro-15-(2,3- dihydro-1H-inden-2-yl)-16,17,18,19,20- pentanor-prosta-5Z,13E-dien-1-oic acid (AL8810, a PGF_2α_ receptor (FP) antagonist, 10 µmol/L). All drugs were added 30 min before the concentration-response curve to noradrenaline.

In another set of experiments, the vasoactive response to the TP receptor agonist (15)-hydroxy-11,9 -(epoxymethano)prosta-5,13-dienoic acid (U46619, 1 nmol/L - 10 µmol/L), PGE_2_ (10 nmol/L –10 µmol/L) or PGF_2α_ (10 nmol/L to 10 µmol/L), was analyzed in endothelium-denuded arteries from 6- and 12-month old rats.

### Prostanoids Production

To measure the release of TxA_2_, PGE_2_ and PGF_2α_, we used specific enzyme immunoassay kits (Cayman Chemical Company, Ann Arbor, MI, USA). The second, third and fourth branches of mesenteric artery were preincubated for 45 min in 200 µl of KHS at 37°C and continuously gassed with a 95% O_2_ - 5% CO_2_ mixture (stabilization period). Afterward, three washout periods of 7 min in a bath of 200 µl of KHS were run before incubation with noradrenaline (10 nmol/L - 0.1 mmol/L. The medium was collected 10 min after the last dose of noradrenaline. The different assays were performed following the manufacturer’s instructions. Results were expressed as pg.ml^−1^ mg wet tissue^−1.^

### Drugs

Drugs used were noradrenaline hydrochloride, acetylcholine chloride, indomethacin, L-NAME (Sigma; St. Louis, MO, U.S.A.), NS-398, SC-560, SQ29548, AH6809, AL8810, PGE_2_, PGF_2α_ (Cayman Chemical Company; Ann Arbor, Michigan, U.S.A.) and U46619 (Calbiochem-Novabiochem GmbH). Stock solutions of acetylcholine was made in distilled water, noradrenaline was dissolved in a NaCl (0.9%)-ascorbic acid (0.01% wv^−1^) solution, indomethacin, SQ29548, PGE_2_ and PGF_2α_ were dissolved in ethanol and AH6809, AL8810 and U46619, which were dissolved in dimethyl sulfoxide. These solutions were kept at −20°C and appropriate dilutions were made on the day of the experiment.

### Statistical Analysis

Noradrenaline, U46619, PGE_2_ and PGF_2α_ contractile responses were expressed as a percentage of the maximum response produced by KCl.

All values are expressed as means±S.E.M. of the number of animals used in each experiment. Results were analyzed using two-way ANOVA for comparison between groups. When ANOVA showed a significant treatment effect, Bonferroni’s post hoc test was used to compare individual means. Differences were considered statistically significant at P<0.05.

## Results

**Figure 1:**
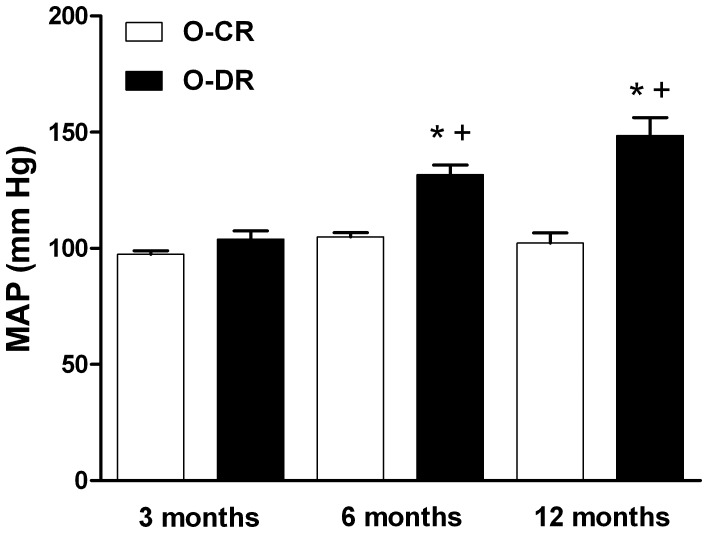
Effect of maternal diabetes on blood pressure levels in the offspring. Mean arterial pressure (MAP) significantly increased from 6 months in the offspring from diabetic mothers (O-DR) compared to control mother offspring group (O-CR). Results are expressed as means±SEM, N = 7–8 animals in each group. ANOVA (two-way): *P<0.05 O-DR *vs*. age-matched O-CR; +P<0.05 6- or 12-month-old O-DR *vs*. 3-month-old O-DR.

O-DR presented higher blood pressure in adulthood. Although the mean arterial pressure of 3-month-old rats was similar in both groups (Figure 1) it was significantly increased in both 6 and 12 months (Figure 1) of age in O-DR compared to O-CR. The heart rate was similar in all O-DR groups compared to their respective age-matched O-CR (results not shown).

**Figure 2:**
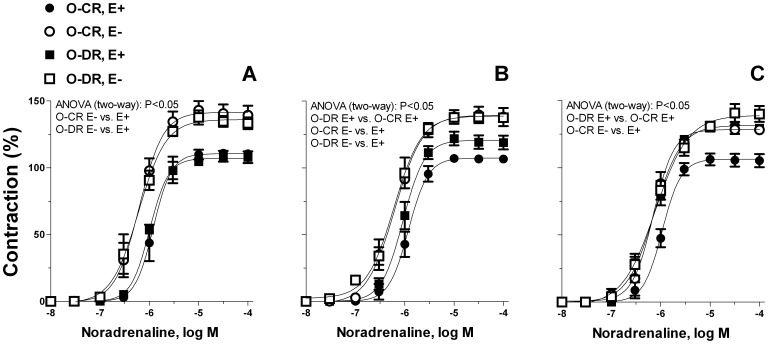
Concentration-dependent contraction to noradrenaline in endothelium-intact (E+) and denuded (E−) mesenteric resistance arteries from 3- (A), 6- (B) and 12-month-old offspring of diabetic (O-DR) and non-diabetic rats (O-CR). Results (mean±S.E.M.) were expressed as a percentage of the initial contraction elicited by KCl. N = 6−7 animals each curve.

KCl (120 mmol/L) evoked similar contractions in vessels from all groups studied (3-month-old rats, O-CR: 3.15±0.04 *vs*. O-DR: 3.19±0.07 mN/mm; 6-month-old rats, O-CR: 3.28±0.12 *vs*. O-DR: 3.19±0.09 mN/mm; 12-month-old rats, O-CR: 3.21±0.02 *vs*. O-DR: 3.25±0.13 mN/mm; ANOVA (two-way), P>0.05). However, exposure to maternal diabetes promoted an increase in the contractile response to noradrenaline in a time-dependent manner in arteries from the offspring (Figure 2). While in arteries from 3-month-old O-DR the noradrenaline-induced contraction remained unmodified (Figure 2A), in both 6- and 12-month-old O-DR this response was increased when compared to age-matched O-CR (Figure 2B and C). In arteries without endothelium the contractile responses produced by noradrenaline were similar in all groups studied (Figure 2).

**Figure 3:**
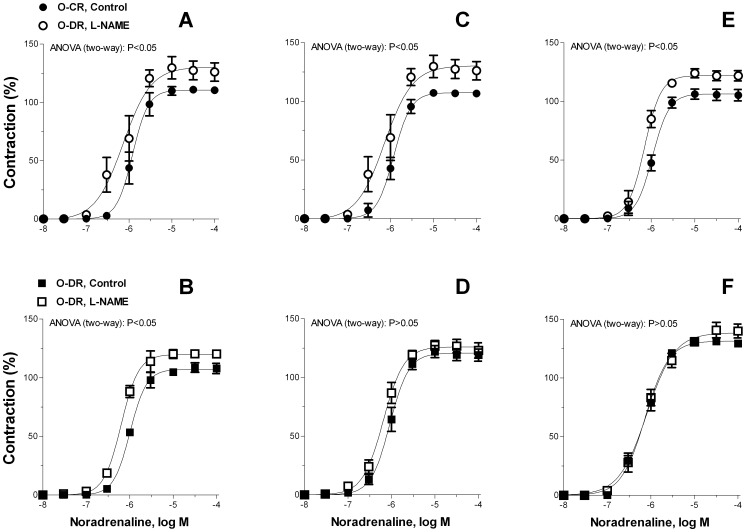
Effect of L-NAME (0.1 mmol/L) on the concentration dependent contraction to noradrenaline in mesenteric resistance segments (A) 3-month-old O-CR, (B) 3-month-old O-DR, (C) 6-month-old O-CR, (D) 6-month-old ODR, (E) 12-month-old O-CR and (F) 12-month-old ODR. Results (mean±S.E.M.) are expressed as a percentage of the initial contraction elicited by KCl. N = 7−8 animals each curve.

To assess the contribution of endothelium-derived nitric oxide to the noradrenaline responses, segments were incubated with the nitric oxide synthase inhibitor L-NAME. This drug similarly increased the response to noradrenaline in arteries from 3-, 6- and 12-month-old O-CR and 3-month old O-DR. (Figure 3). In arteries from 6- and 12-month-old O-DR L-NAME failed to produce significant increases of noradrenaline-induced contraction (Figure 3).

**Figure 4:**
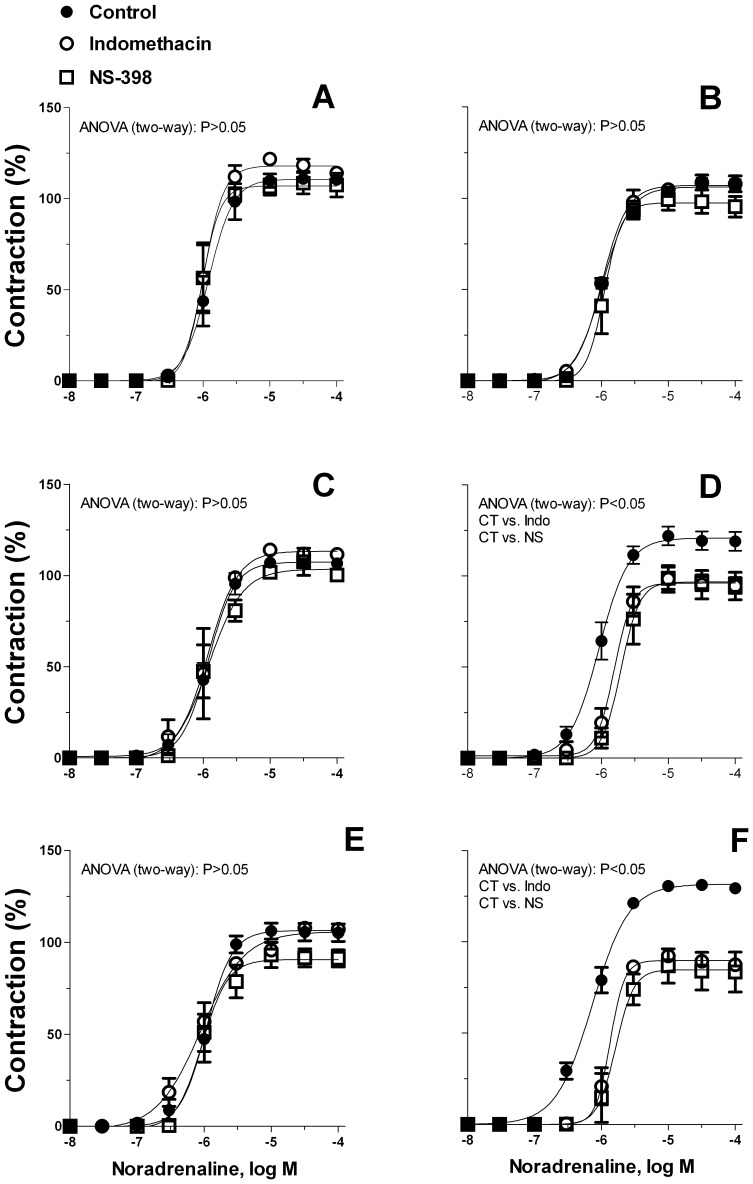
Effect of indomethacin (Indo, 10 µmol/L) or NS-398 (NS, 10 µmol/L) on the concentration dependent contraction to noradrenaline in mesenteric resistance segments (A) 3-month-old O-CR, (B) 3-month-old O-DR, (C) 6-month-old O-CR, (D) 6-month-old ODR, (E) 12-month-old O-CR and (F) 12-month-old ODR. Results (mean±S.E.M.) are expressed as a percentage of the initial contraction elicited by KCl. N = 6−7 animals each curve.

In mesenteric resistance arteries from 6- and 12-months old rats, incubation with either the unspecific COX inhibitor indomethacin or the specific COX-2 inhibitor NS-398 decreased the contractile response to noradrenaline only in O-DR (Figure 4C and D). In 3-month-old O-CR and O-DR, the contraction to noradrenaline did not change in presence of indomethacin or NS-398 (Figure 4A and B). Preincubation with the specific COX-1 inhibitor SC-560 did not alter the contraction to noradrenaline in any experimental group (results not shown).

**Figure 5:**
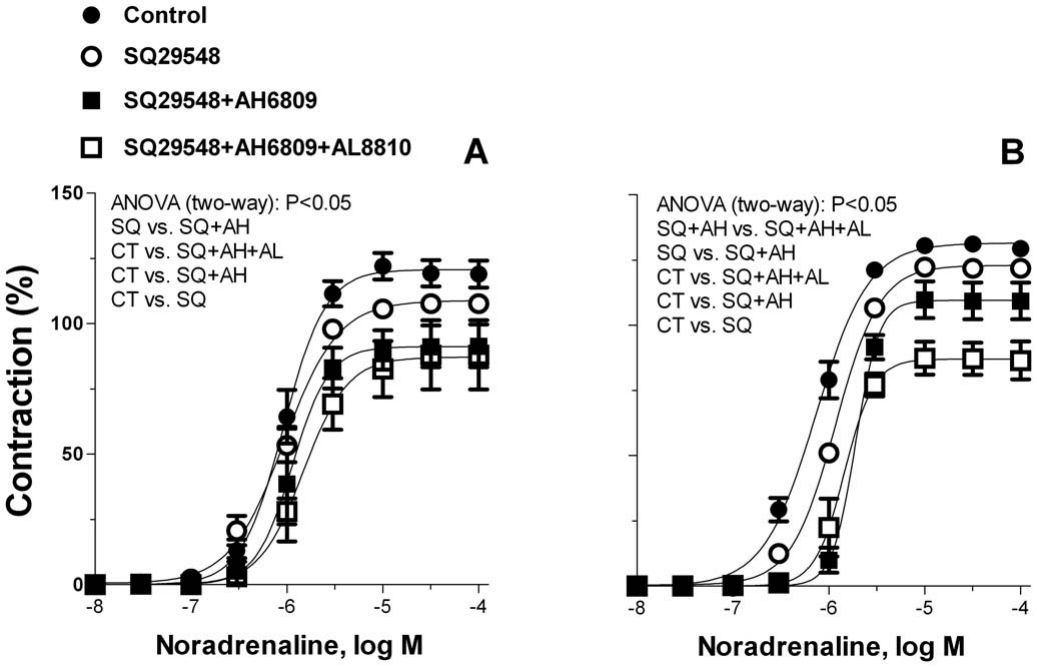
Effect of SQ29548 (SQ, 1 µmol/L) alone or in association with AH6809 (AH, 30 µmol/L) or AH6809 plus AL8810 (AL, 10 µmol/L) on the concentration-dependent contraction to noradrenaline in mesenteric resistance segments from 6- (B) and 12-month (mo)-old (C) diabetic (O-DR) offspring rats. Results (mean±S.E.M.) are expressed as a percentage of the initial contraction elicited by KCl. N = 6−7 animals in each group.

To identify the contractile prostanoid involved in noradrenaline responses, arteries from 6- and 12-month-old O-DR were incubated with the TP receptor antagonist SQ29548, the EP_1_, EP_2_ and EP_3_ antagonist AH6809 and the FP antagonist AL8810. In 6-month-old O-DR, SQ29548 decreased contraction to noradrenaline, but its effect was lower than to those obtained with NS-398 (Compare figure 4D with 5A). When co-incubated with SQ29548, AH6809 reduced the response to noradrenaline to similar values to those obtained with NS-398. AL8810 failed to decrease the effect of SQ29548 plus AH6809 (Figure 5A). In arteries from 12-month-old O-DR, SQ29548 partly decreased contraction to noradrenaline (Figure 5B). In arteries from this group, although co-incubation with AH6809 have increased the effect of SQ29548, an additional decreased of the noradrenaline response was observed when arteries were treated with SQ29548 in combination with AH6809 and AL8810 (Figure 5B).

**Figure 6:**
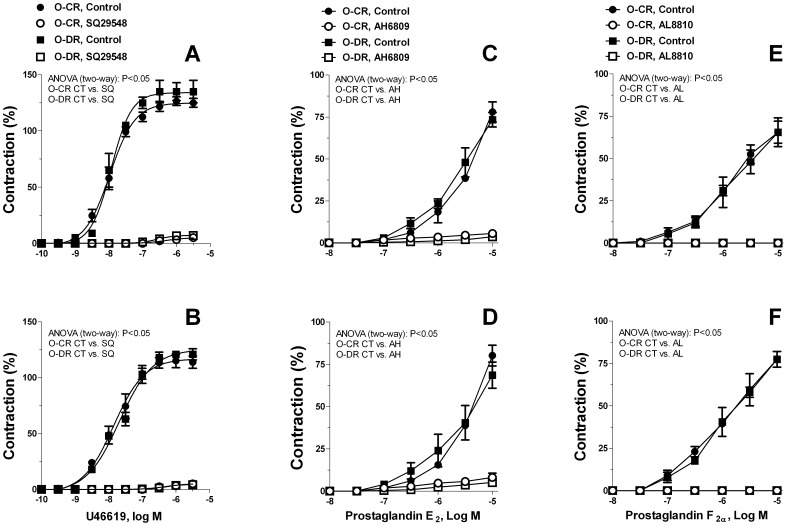
Concentration-dependent contraction to U46619 (A and B), PGE_2_ (C and D) and PGF_2α_ (E and F) in endothelium-denuded mesenteric resistance arteries from 6- (A, C and E) and 12-month-old (B, D and F) offspring of diabetic (O-DR) and non-diabetic rats (O-CR). Curves to U46619, PGE_2_ and PGF_2α_ were performed in absence (Control, CT) and in presence of SQ29548 (SQ), AH6809 (AH) or AL8810 (AL), respectively. Results (mean±S.E.M.) are expressed as a percentage of the initial contraction elicited by KCl. N = 4−6 animals in each group.

U46619, PGE_2_ and PGF_2α_ caused cumulative concentration-dependent contractions in quiescent resistance arteries. These contractions were not significantly different between arteries of O-CR and O-DR (Figure 6). Contraction to U46619, PGE_2_ and PGF_2α_ were inhibited by SQ29548, AH6809 and AL8810, respectively (Figure 6).

**Figure 7:**
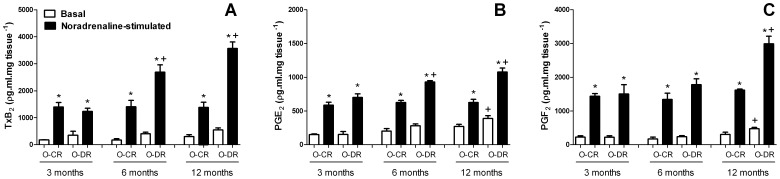
Basal and noradrenaline-stimulated production of thromboxane B_2_ (TxB_2_, A), prostaglandin E_2_ (PGE_2_, B) and prostaglandin F_2α_ (PGF_2α_, C) in mesenteric resistance segments from 3-, 6- and 12-month-old control (O-CR) and diabetic (O-DR) offspring rats. N = 6−7 animals in each group. ANOVA (two-way): *P<0.05 noradrenaline-stimulated vs. basal; +P<0.05 O-DR vs. age-matched O-CR.

In mesenteric resistance arteries from all groups, noradrenaline increased the release of TxB_2_, PGE_2_ and PGF_2α_ (Figure 7). The noradrenaline-stimulated TxB_2_ levels were higher in arteries from both 6- and 12-month-old O-DR compared to their respective age-matched O-CR (Figure 7A). In arteries from 6- and 12-month-old O-DR, the levels of noradrenaline-stimulated PGE_2_ were higher compared to O-CR (Figure 7B and C). The noradrenaline-stimulated PGF_2α_ levels were higher only in arteries from 12-month-old O-DR (Figure 7C).

## Discussion

Results presented here demonstrate that *in utero* exposure to maternal hyperglycaemia in rats increases the participation of COX-2-derived TxA_2_, PGE_2_ and PGF_2α_ on contraction to noradrenaline. This participation, associated to decrease in nitric oxide modulation, might help to explain the greater noradrenaline responses observed in mesenteric resistance arteries from 6- and 12-month-old offspring of diabetic rats, which could partially contribute to the development of hypertension and vascular disease in these rats.

The intrauterine exposure to maternal hyperglycaemia is a significant risk factor for development of metabolic syndrome components, including glucose intolerance, insulin resistance and hypertension [5], [24]. The mechanisms of this hyperglycemia-programmed hypertension are complex and involve renal, neural and vascular factors [1], [2], [4], [8], [10]. One of the most remarkable vascular changes in rats born from diabetic mothers is the reduction of endothelium-dependent relaxation [2], [8], [12]. Ingram *et al*. [25] reported that *in utero* exposure to diabetic environment severely results in general decline in proliferative capacity of fetal circulating endothelial progenitor cells, producing a decrease in the number of endothelial cells and their premature senescence. These alterations may predispose infants born from diabetic mothers to develop endothelial dysfunction and ultimately cardiovascular disease.

In a recent study we reported that offspring of diabetic rats manifest glucose intolerance, insulin resistance and hypertension at 6 and 12 months of age [9]. In addition, resistance arteries from these rats also displayed impaired endothelial function, associated with increased COX-2-derived vasoconstrictor prostanoids formation [9]. Several studies have demonstrated a correlation between endothelial dysfunction and the hyperreactivity to the vasoconstrictor agents [15], [26]–[28]. Herein, we have provided clear evidence that compared to O-CR, mesenteric resistance arteries isolated from O-DR is indeed hyperreactive to noradrenaline. This effect is endothelium-dependent, since in endothelium-denuded arteries noradrenaline contraction was similar between O-CR and O-DR. The unmodified K^+^-induced contraction in resistance arteries from O-DR suggests no alteration in contractile ability of the smooth muscle. Our results also show that the increased response to noradrenaline in arteries from O-DR correlates with changes of blood pressure in these rats, suggesting that this mechanism could be responsible for the enhanced arteriolar tone and consequently elevated systemic blood pressure in these rats.

The next objective was to determine possible quantitative or qualitative differences in the participation of endothelial factors in this contractile response in both O-CR and O-DR. Nitric oxide synthesis inhibition (L-NAME) increased the vasoconstriction to noradrenaline in arteries from all O-CR groups, while in 6- and 12-month-old O-DR it failed to produce significant increases of noradrenaline-induced contraction. This suggests a reduction on nitric oxide release/bioavailability in arteries from 6- and 12-month-old O-DR, which in turn could contribute to the augmented noradrenaline response in these rats.

COX-1 and COX-2 activity may contribute to underlying vascular hyperreactivity in some cardiovascular disease models [15], [18], [29]–[32]. Many studies have shown that COX-2 is induced during some inflammatory process and prostanoids produced by this isoform are responsible for many inflammatory signs [13], [33]. In this sense, COX-2-derived prostanoids have been shown to be associated with the development of vascular complications under insulin resistance conditions and cardiovascular risk [13], [14], [16], [20], [34], [35]. Results presented here and previously [9] provide several lines of evidence suggesting that COX-2 contributes to the hyperreactivity to noradrenaline in mesenteric resistance arteries from O-DR. First, the immunoblot analysis clearly shows that COX-2, but not COX-1, is up-regulated in arteries from O-DR [9]. Second, the time course of COX-2 up-regulation [9] coincides with the appearances of hyperreactivity to noradrenaline in O-DR (6 and 12 months of age). Third, selective inhibition of COX-2 activity with indomethacin or NS-398, but not the inhibition of COX-1 with SC-560, significantly reduced the noradrenaline-induced contractile responses in arteries isolated from 6- and 12-month-old O-DR, whereas these drugs did not affect the contractions in arteries from O-CR. Taken together, these results suggest that the selective COX-2 up-regulation in mesenteric resistance arteries is responsible for the contractile hyperreactivity in offspring of diabetic rats.

Data from our group [9], [19] and others [15], [35], [36], [37] have showed that COX-2 up-regulation is associated with increased production of the vasoconstrictor prostanoids, such as TxA_2_. This denotes that the increased production of TxA_2_ is one potential mechanism mediating COX-2-induced vascular hyperreactivity. Indeed, TP receptor blockade by SQ29548 diminished the vascular smooth muscle hyperreactivity in both 6- and 12-month-old O-DR. In addition, noradrenaline-stimulated release of TxB_2_ (the TxA_2_ metabolite) was increased in arteries from those rats. However, it should be noted that in arteries from 6 and 12-month-old O-DR the decrease in noradrenaline-induced contraction produced by SQ29548 was lower than that observed in the presence of indomethacin or NS-398. In mesenteric arteries from 6-month-old O-DR, reduction in the noradrenaline response to the level produced by COX-2 inhibition occurred when these vessels were preincubated with SQ29548 plus EP receptor blocker, AH6809. Among 12-month-old O-DR the combined treatment of SQ29548 plus AH6809 and AL8810 (a FP receptor antagonist) was required to reduce noradrenaline response to the level produced by NS-398 or indomethacin. This indicates that in resistance arteries from 6-month-old O-DR the increase in noradrenaline-induced contraction was produced by an increase in both TxA_2_ and PGE_2_ while in arteries from 12-month-old O-DR the hyperactivity to noradrenaline was produced by an increase in PGE_2_, TxA_2_, and PGF_2α_ release. Consistent with these functional findings, in addition to TxA_2_, noradrenaline-stimulated PGE_2_ release was also higher in arteries from 6- and 12-month-old O-DR. Besides these two prostanoids, noradrenaline-stimulated PGF_2α_ was also increased in arteries from 12-month-old O-DR. The fact that exogenous PGE_2_, PGF_2α_ or TP receptor agonist U46616 administration have produced similar contraction in endothelium-denuded arteries in all groups studied discard the possibility that EP, FP or TP receptor initiated signaling mechanisms are altered in mesenteric resistance arteries from O-DR.

As reported by several studies, increased activation of TP, EP and/or FP receptors represents a key mechanism to increased vascular tone and blood pressure in several models of cardiovascular disease, including hypertension and diabetes [38]–[46]. Rutkai *et al*. [38] demonstrated that EP receptor activation in resistance arteries may contribute to the development of high blood pressure in type-2 diabetic mice. In these animals the augmented pressure- and angiotensin II-induced arteriolar tone, as well as the elevated systolic blood pressure were normalized by the EP antagonist, AH6809. Similarly, Guan *et al*. [39] demonstrated that EP receptor activation contributes to hypertension in two well-established experimental models: SHR and chronic angiotensin II-infused mice. Respect to TxA_2_, various studies have reported that TP receptor activation contributes to the severity of blood pressure elevation as well as cardiac hypertrophy and increased vascular responsiveness in hypertensive rats [40], [41], [42], [43]. PGF_2α_ has also been involved in the pathogenesis of hypertension. Tian *et al*. [44] demonstrated that COX-2-derived PGF_2α_ contributes to endothelial dysfunction and elevation of blood pressure in renovascular hypertensive rats. In addition, Yu *et al*. [45] have also reported that FP receptor deletion decreased blood pressure in mice, indicating that PGF_2α_ may act via FP receptor to modulate blood pressure regulation.

Results presented here provide evidences of participation of COX-2-derived TxA_2_, PGE_2_ and PGF_2α_ in the altered regulation of mesenteric arterial responsiveness in offspring of diabetic rats. As abnormal vascular reactivity may contribute to the etiology of hypertension [47]–, our results suggest a crucial role for these COX-2-derived prostanoids in elevating vascular resistance and systemic blood pressure in offspring of diabetic rats. However, these results should be careful interpreted once blood pressure was not analyzed after chronic COX-2 inhibition or TP, EP and FP receptors blockade. Further studies have yet to be performed to demonstrate this effect in offspring of diabetic rats.

Collectively, our results suggest that a) age-dependent hyperreactivity to noradrenaline is present in mesenteric resistance arteries of adult offspring of diabetic rats, b) it may result from a reduction on nitric oxide release/bioavailability and increased TxA_2_, PGE_2_ and PGF_2α_ vasoconstrictor prostanoid releasing, and c) the mechanisms underlying this hyperreactivity may involve up-regulation of COX-2 [9]. However, further studies need to be carry out to determine whether and for what extent changes in mesenteric prostaglandin synthesis might contribute to blood pressure alterations in these rats. Nevertheless, changes in COX-dependent modulation of contractile response should be taken into consideration in future studies with offspring born from diabetic mothers.
